# Diagnosis and Treatment of Odontogenic Cutaneous Sinus Tracts in an 11-Year-Old Boy: A Case Report

**DOI:** 10.1097/MD.0000000000003662

**Published:** 2016-05-20

**Authors:** Ke Chen, Yun Liang, Huacui Xiong

**Affiliations:** From the Department of Stomatology of Guangzhou Women and Children's Medical Center (KC,YL,HX); and Medicine School of Jinan University (YL), Tianhe District, Guangzhou, Guangdong, China.

## Abstract

Odontogenic cutaneous sinus tracts (OCSTs) are generally primarily misdiagnosed and inappropriately treated by virtue of their rarity and the absence of dental symptoms. Accurate diagnosis and treatment and the elimination of the source of infection can reduce the incidence of complications and relieve the pain of the patient.

In this case report, we present the case of an 11-year-old patient with an apparent abscess but an unobvious draining sinus tract in his left cheek. Intraorally, a glass-ionomer-cement filling on the occlusal surface of the left mandibular first molar (tooth 36) was noted. Radiographic examination revealed a radiopaque mass inside the crown and pulp chamber and an irregular, radiolucent periapical lesion surrounding the distal root apex. He was diagnosed with an OCTS secondary to a periapical abscess of tooth 36. Precise root canal therapy (RCT) and chronic granuloma debridement was performed; 6 months later, the abscess and sinus had healed completely, and the periapical lesion had resolved.

Odontogenic cutaneous sinus tracts are uncommon in the clinic. This case report reminds us of the significance of OCSTs and provides some implications for their diagnosis and treatment.

## INTRODUCTION

Odontogenic cutaneous sinus tracts (OCSTs) are uncommon, but they are generally initially misdiagnosed and ineffectively treated.^1^ OCSTs on the facial and cervical skin have been to progress from dental pulp necrosis and chronic periapical periodontitis.^2^ Extraoral sinus tracts mostly appear in the mandibular angles, chin, and cheeks. Extraoral fistulas are typically characterized as erythematous, symmetrical, crusting, smooth, and nontender nodules with periodic drainage.^3^ However, they pose a diagnostic challenge because the lesions can be confused with pyogenic granuloma, foreign body reaction, deep fungal infection, squamous cell carcinoma, or osteomyelitis.^4^ Patients often fail to realize the odontogenic etiology and most likely seek help from dermatologists due to a consistent facial nodules but no dental symptoms. It has been evaluated that half of the patients with extraoral sinus tracts undergo numerous dermatological surgical operations and long-term antibiotic therapy—even radiotherapy, electrodessication, or cancer-directed therapy—before the true diagnosis is established, resulting in relapse of the basic problem and the chronic persistence of the lesion.^5^ Treatment of these fistulas involves either RCT of healable teeth or extraction of unrestorable teeth. Considering the scarceness and diagnostic predicament of OCSTs, we report a case of an 11-year-old male adolescent.

Written informed consent was obtained from the parents of the patient.

## CASE REPORT

An 11-year-old boy was referred to the Department of Stomatology, Guangzhou Women and Children's Medical Center, in Guangzhou, P.R. China, to confirm a probable dental cause of a skin lesion. The patient's chief complaint was the presence of a nonhealing extraoral swelling on his left cheek for 6 months, which periodically discharged pus and gradually grew larger. During history taking, his parents stated that a localized swelling abruptly arose on the boy's left cheek without pain, fever, or other discomfort. They also disclosed that he had been submitted to dermatological surgery for removal of a cutaneous lesion and had gone multiple regimens of antibiotic therapy, but the persistence of the lesion had brought him great suffering. Therefore, he was referred to our Conservative Dentistry and Endodontics Department.

**FIGURE 1:**
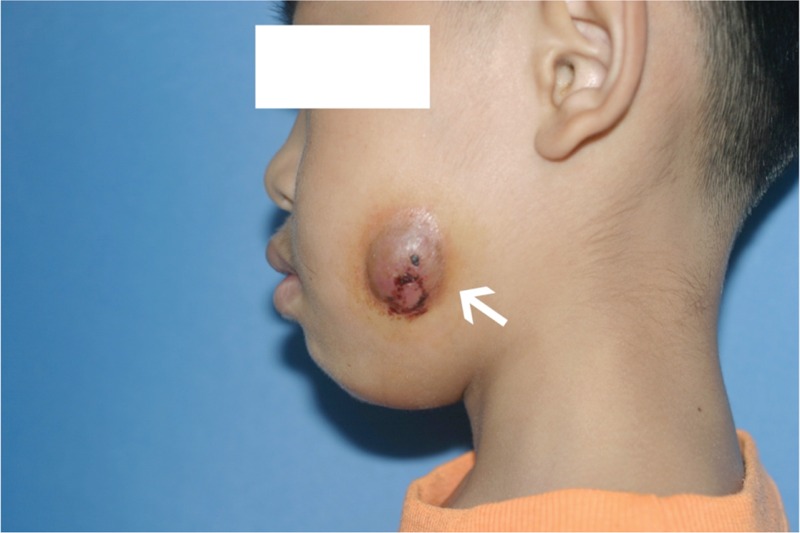
Preoperative extraoral appearance of abscess measured 2.5 cm in diameter with draining lesion on the left cheek (indicated by the arrow).

**FIGURE 2:**
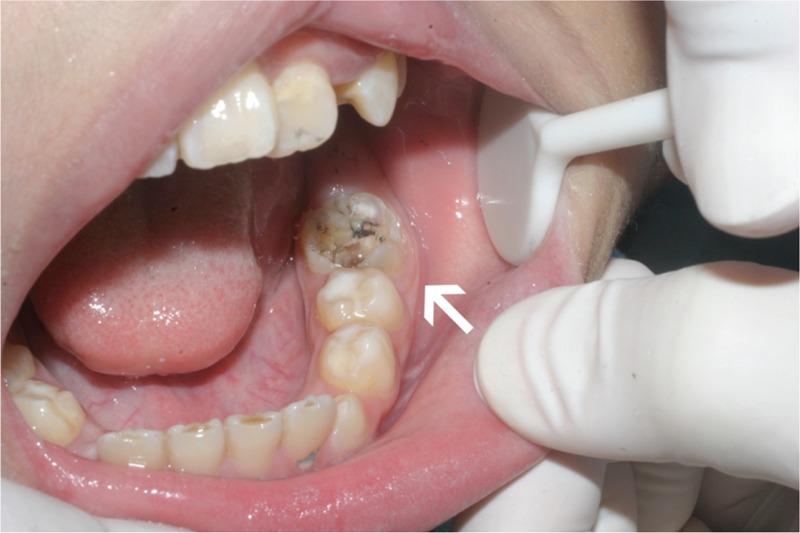
Intraoral view showed the left mandibular first molar (tooth 36) after removing the filling (indicated by the arrow). The gum and apical mucosa of were normal without red and swollen.

On extraoral examination, a reddish abscess 2.5 cm in diameter with an unobvious sinus tract on his left cheek 2 cm above the inferior border of the mandible was observed (Figure 1). On palpation, the swelling was fluctuant, and it mildly ached after pressure; concurrently, palpation of the surrounding tissue produced yellow pus continuously. Intraorally, a glass–ionomer–cement filling on the occlusal surface of tooth 36 was noted. The tooth was tender to slight percussion and painful, and it did not respond to electrical pulp testing or heat testing. Periodontal probing around the tooth discovered a pocket depth within physiological limits. The vestibular mucous membrane corresponded to the apex of the root, and the periodontal tissue was normal without an intraoral sinus (Figure 2).

**FIGURE 3:**
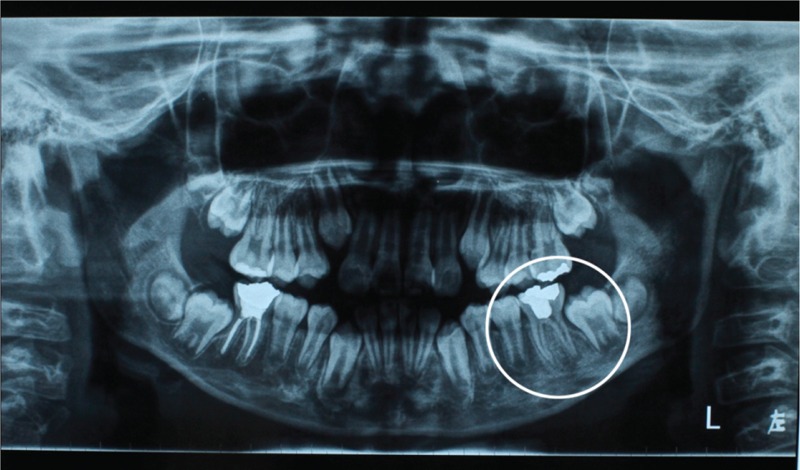
Preoperative radiograph revealed a periapical radiolucent area related to the distal root apex.

Nucleic acid quantitative detection of *Mycobacterium tuberculosis* and tuberculosis antibody detection were performed to exclude other diseases, and both results were negative. In addition, bacterial cultures of pus from the lesion proved that the infection rate of specific aerobic and anaerobic bacteria were 0%. The preoperative radiographic examination revealed a radiopaque mass inside the crown and pulp chamber, an irregular periapical radiolucent but non-noticeable area associated with the distal root apex (Figure 3). Based on the aforementioned findings, we could draw a preliminarily conclusion that the patient had an odontogenic subcutaneous abscess with a cutaneous fistula on the left cheek secondary to chronic periradicular periodontitis of tooth 36.

**FIGURE 4:**
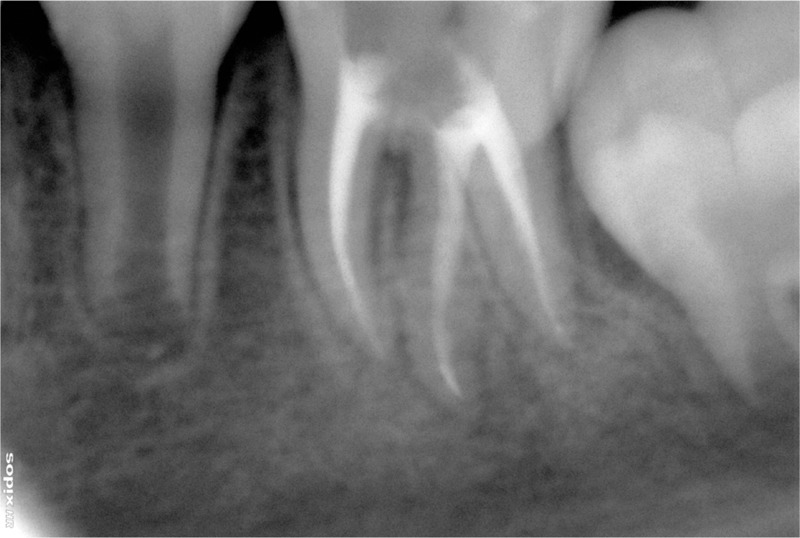
. Postoperative radiograph revealed tooth 36 after root canal therapy (RCT). The periapical radiolucent area around the distal root apex has reduced. RCT = root canal therapy.

**FIGURE 5:**
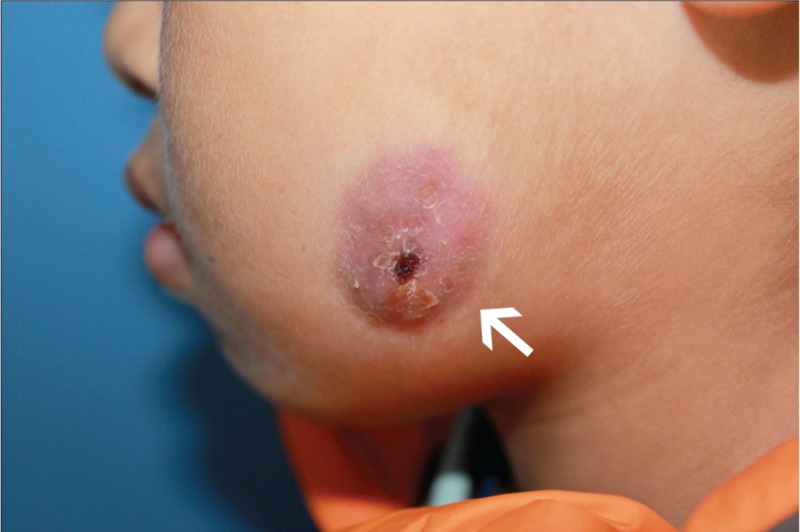
Postoperative appearance 3 weeks later after RCT, red granulation tissue still existed (indicated by the arrow). RCT = root canal therapy.

**FIGURE 6:**
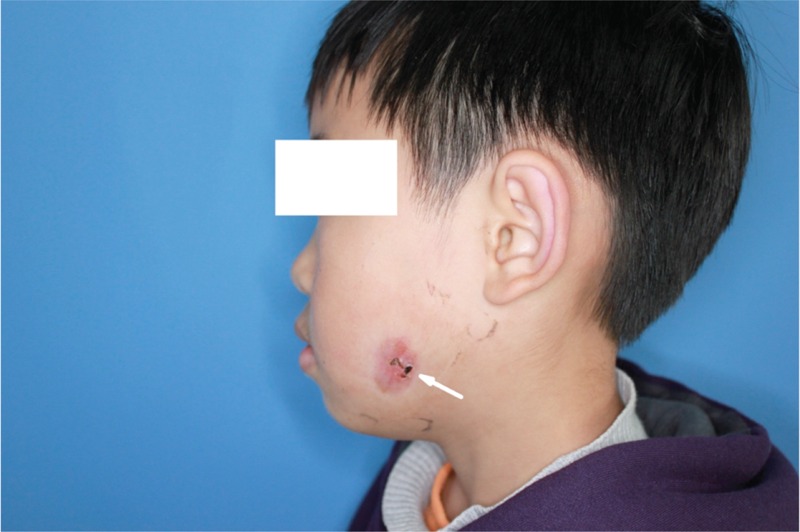
Postoperative appearance 2 weeks later after granuloma debridement, the lesion almost healed (indicated by the arrow).

**FIGURE 7:**
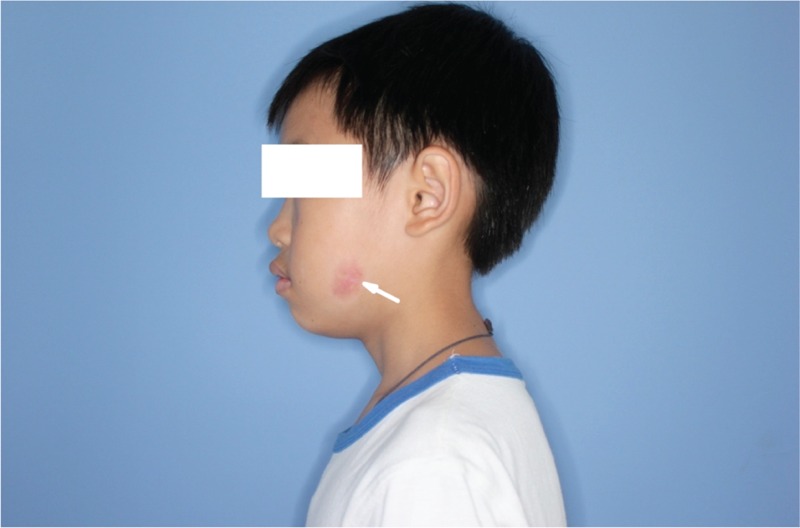
Postoperative appearance 6 months later after granuloma debridement showed complete healing of the abscess with slightly hyperpigmented region (indicated by the arrow).

**FIGURE 8:**
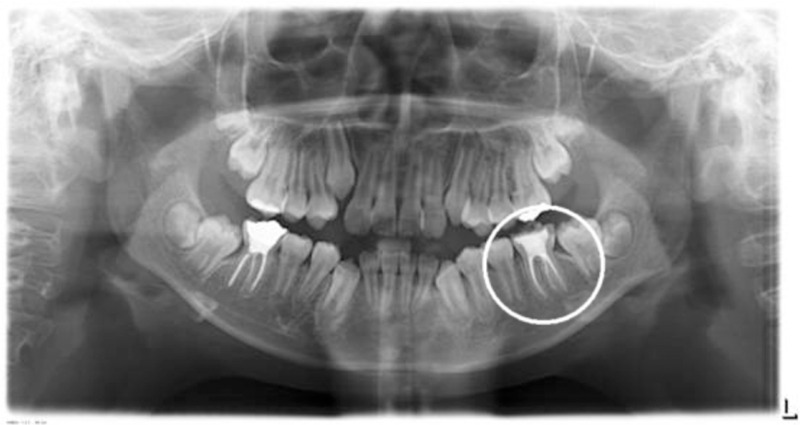
Radiographic appearance after 6 months showed the lesion disappeared.

After obtaining their informed consent, the root was treated with RCT. A certain dark red hemorrhaging occurred after a trapezoidal access opening was established; afterward, we cleaned all 4 of the root canals of tooth 36 using ISO size #8 or #6 stainless steel K-files (Dentsply, York, PA) and 3% hydrogen peroxide (HengJian Co., Ltd., Jiangmen, Guangdong, China), kept the pulp chamber patulous, and performed abscess incision drainage, making an appointment for him 5 days later. The patient returned with a remarkably reduced abscess. Unlike the first presentation, his parents were full of expectation and positivity. Hence, after situating a hygenic rubber dam (Coltene, Feldwiesenstrasse, Altstatten, Switzerland), the root canals were cleaned and shaped with Protaper (Dentsply, York, PA) Ni-Ti rotary instruments, using a crown-down technique under irrigation with 3% sodium hypochlorite solution (Nippon Shikayakuhin Co.,Ltd., Shimonoseki, Honshu, Japan) and 3% hydrogen peroxide to S2, up to a working length of 19.5 mm for 4 canals, namely the mesiobuccal (MB), mesiolingual (ML), distobuccal (DB), and distolingual (DL) canals. Working lengths were measured with a Raypex5 electronic apex-locator (VDW, Munich, Bavaria, Germany). The canals were dressed with Vitapex (Morita, Osaka, Honshu, Japan) medicaments. At the third visit, 2 weeks later, the OCST was closed, and the root canals were obturated with gutta-percha points (Dentsply, York, PA) and Cortisomol sealer (Morita, Osaka, Honshu, Japan) using the cold lateral condensation method. Postoperative radiography indicated successful obturation (Figure 4). At the next visit, approximately 3 weeks later, the abscesses became flatter after RCT; nonetheless, red granulation tissue was evident on the cheek (Figure 5). Subsequently, chronic granuloma debridement of the lesion under general anesthesia, along with Scandonest local anesthesia, was performed. The granulation area turned into smooth tissue, and the lesion was almost healed 2 weeks later (Figure 6). Six months later, the defect on the cheek had eventually become no different from the surrounding tissue, leaving only a slightly hyperpigmented region of the skin (Figure 7). The periapical lesion disappeared on the radiographic examination, demonstrating satisfying recovery (Figure 8).

## DISCUSSION

If a draining lesion on the facial or cervical area, an endodontic origin should be considered a priority in differential diagnosis.^6^ Other etilologies consist of pyogenic granuloma, foreign body reaction, deep fungal infection, squamous cell carcinoma, osteomyelitis, and so on.^4^ OCSTs are typically caused by periapical infections around the root apices as a result of pulpal necrosis, nearby caries, pericoronitis, or traumatic injury.^7^ This chronic process slowly evolves through the cancellous alveolar bone, following the path of least resistance until it perforates the cortical plate of the mandible and forms an subperiosteal abscess.^8^ OCSTs have a greater likelihood of occurring than intraoral sinus tracts if the apices of the teeth are superior to the maxillary muscle attachments or inferior to the mandibular muscle attachments.^9^ Cutaneous fistulas then arise from the spread of infection into the surrounding soft tissues. When OCSTs occur, they develop from a mandibular abscess and drain onto the chin or the submandibular area (80%). In this case, a ruddy abscess without an obvious sinus tract on the patient's left cheek 2 cm above the inferior border of the mandible attracted our attention at the first presentation. OCSTs can also involve the nose, nasolabial folds, and inner canthi of the eyes.^10^

A sinus tract prevents swelling or pain from increased pressure because it provides drainage from the primary odontogenic site.^9^ However, in the case reported here, what could be seen on the patient's cheek was initially swelling with fluctuation but not an apparent sinus tract. A possible reason might be that the patient's immune system was immature, so his condition worsened. Serious or serve onset and the cutaneous tract and lesion are only rarely associated with symptoms from the oral cavity. Symptoms from the teeth only present in 50% of patients, which could explain why patients frequently consult with a physician first for help.^5^ Furthermore, OCSTs do not always occur near the original odontogenic cause. These 2 elements united may engender a postponed in diagnosis.^11^ Regrettably, delays in corroborating the factual diagnosis often lead to unnecessary antibiotic treatment and redundant surgical interventions. Appraisal of OCSTs must start with an intensive medical and clinical history, and odontogenic infection must be borne in mind in the differential diagnosis of such lesions on the face or neck.^12^ Hence, the clinician should pay extraordinary attention to oral clinical conditions, such as caries, deficient restorations, and periodontal conditions.^13^

Because major infections are polymicrobial, microbiologic culture and sensitivity testing of the sinus tract pus should be performed to identify the microbial flora. Culture should also be performed for suspicion of fungal or syphilitic infections.^9^ In the clinical case described here, bacterial cultures of exudate from the lesion certified that *M tuberculosis* was negative; moreover, no specific aerobic and anaerobic pathogens were found. In addition, radiographic findings are also important for the diagnosis. In this case, panoramic radiography showed a periapical radiolucency connected to a suspected tooth, in which a pulp sensibility test was unresponsive. On the assumption that the relevant tooth cannot be ascertained by panoramic and periapical radiography, the tracking of the fistula can guide the ultimate diagnosis.^14^

Eradication of the original source of infection is most important for the treatment of OCTSs, by means of nonsurgical RCT, sometimes complemented by surgery or dental extraction.^15^ In this case, prominent reduction of the abscess after nonsurgical RCT validated the primary dental origin of the skin lesion. Automatic closure of the tract should be anticipated within 5 to 14 days after RCT. Surgical excision of the cutaneous sinus is necessary if the fistula does not terminate, to eliminate the inflammatory tissue thoroughly, including apical debridement, apicoectomy, and fistula debridement.^16^ Eventually, in our case, chronic granuloma debridement of the lesion was performed complementally to eliminate infection completely, as mentioned above; thereafter, the lesion on the cheek recovered entirely leaving only a slight hyperpigmented region, and the periapical lesion disappeared on the radiographic examination.

This case emphasizes that maxillary or mandibular dental abscesses are a common cause of OCTSs. It also indicates the usefulness of microbiologic culture before administering antibiotic treatment or performing a surgical intervention.^14^ Comprehensive learning of both normal and abnormal root canal morphology can play a crucial role in outstanding endodontic treatment.^17^

## CONCLUSION

Odontogenic cutaneous sinus tracts (OCSTs) of dental origin are primarily misdiagnosed generally due to their rarity. Although the most considerable reason for a recurrently putrefying cutaneous sinus tract on the facial and cervical skin is persistent dental infection, chronically draining sinus tracts of the face and neck continue to defy diagnosis. Further confusion for clinicians arises because dental symptoms do not regularly occur. Worthless treatment and healing failure can be attributed to misdiagnosis of an odontogenic reason; therefore, obtaining an accurate diagnosis, providing appropriate clinical care, and extinguishing the source of infection are of great seriousness.

In conclusion, due to their rare occurrence, the diagnosis of odontogenic OCTSs might become easier when clinicians are aware of the probability of a dental origin. A rigorous and exact diagnosis relies on concerted referrals and cooperation among physicians, dermatologists, surgeons, and dentists. Finally, integrated learning and the guaranteed technique of a dentist are as important as a correct diagnosis, promoting efficient treatment, maximizing patient contentment, and aesthetic appearance and decreasing the likelihood of additional complications. This case report reminds us of the significance of OCTSs and provides some implications for their diagnosis and treatment.
